# The Design and Development of the Internet-Based System for Testing and Analyzing the Psychological and Physiological Responses During Creative Learning

**DOI:** 10.3389/fpsyg.2022.886972

**Published:** 2022-05-13

**Authors:** Junhui Wang

**Affiliations:** School of Educational Information Technology, South China Normal University, Guangzhou, China

**Keywords:** creative learning, psychological and physiological information, virtual instrumentation, data collecting, galvanic skin response, cognitive

## Abstract

An Internet-based system for testing and analyzing psychological and physiological responses during creative learning was developed using virtual instruments based on microcomputer interfaces and network communication technologies. The system can be used to study and evaluate innovative learning processes and performance at the psychological and physiological levels. This paper presents the design, implementation, and application of the system.

## Introduction

Creative learning is becoming increasingly important in contextual education and twenty first century skills; creativity is one of the skills that keep individuals abreast of current, rapidly evolving technologies and functions as one of the priority skills.

The existing literature shows that the effect of stress on creative learning performance is an inverted U-shaped (curved) relationship. This suggests that a certain level of stress helps learners maintain focus on creative learning tasks through arousal. A review of previous research on the relationship between stress and creativity confirms the existence of negative, positive, and curvilinear relationships between the two. Cognitive, affective, and behavioral engagement are important processes for creativity (Drazin et al., 1999), and moderate levels of activation lead to maximum creativity (Byron et al., 2010).

There has been consistent research on the effect of stress on creative learning task performance, but previous studies have not reached a majority consensus on this relationship. Some studies insist that stress has a positive effect on creative learning task performance; however, others even suggest opposite results or even a curvilinear relationship (Jamal, 1984, 1990). Sometimes, these contradictory results arise from a focus on different types of stress (e.g., challenge stress and hindrance stress) or the difficulty level of the task.

An alternative account of the relationship between stressors and creativity has been proposed by some researchers (Avey et al., 2012), namely, that the relationship between stress and creativity is curvilinear (Yerkes and Dodson, 1908). Activation theory (Gardner, 1986) suggests that stress can improve performance to some extent, but too much activation can limit performance, especially for complex tasks such as creative tasks. Individuals are likely to be most creative at moderate levels of activation. Moderate levels of activation increase task engagement and lead to optimal use of cognitive resources by reducing negative affect (Gardner, 1986; Baer and Oldham, 2006). Conversely, too little or too much activation may bring about a lack of engagement and cognitive interference that may interfere with performance on cognitively demanding tasks. Given that cognitive, emotional, and behavioral engagement is an important process for creativity (Drazin et al., 1999), moderate activation leads to maximum creativity (Byron et al., 2010). Furthermore, most studies on challenge stress have analyzed subjects’ perceived levels of stress, but physiological approaches have not been widely tried.

Stress in creative learning can be measured both psychologically and physically. Psychological assessment of stress responses can be measured and assessed in terms of perceptual, behavioral, and physical responses. The assessment of perceived responses to stressors involves subjective estimates and perceptions. Self-reported questionnaires have been the most commonly used tool to measure stress (Cohen, 1988). For a long time, research and evaluation of learners’ stress, emotions, and academic performance during creative learning still mostly use questionnaires and expert scoring, and there are still large gaps in the collection and evaluation of student information, ignoring information on emotions, mental activity, and changes in attention during creative learning, resulting in a lack of objective evidence that is difficult to control and quantify. The physiological response to stress has two components: a physiological response indicating central-autonomic activity and a biochemical response involving changes in the endocrine and immune systems (Rosch, 1997).

Stress induces changes in autonomic physiological function (Van de Kar and Blair, 1999). GSR is a measure of skin electrical resistance. The transient increase in skin conductivity is proportional to sweat secretion (Darrow, 1964). Thus, whenever a person is stressed, sweat gland activity is activated and increases skin conductivity. Since sweat glands are also under the control of the SNS, skin conductivity functions as an indicator of sympathetic activation due to stress response.

Many attempts have been made to detect human psychological and physiological states by employing various physiological sensors (Andreassi, 2007; Enderle and Bronzino, 2012). Brain waves (or electroencephalogram; EEG) and heat rate variability (HRV) have been frequently introduced to assess human psychophysiological states, while other bioelectrical signals, such as skin temperature (Nozawa and Uchida, 2009) and skin conductivity (or electrical skin activity; EDA) (Healey and Picard, 2005), have recently been included in the list of physiological measurements.

According to modern cognitive psychology and psychophysiology, by observing and measuring various physiological responses of learners during creative learning, information on emotional responses, attentional changes, and cognitive processing during creative learning can be captured and used as objective evidence for creative learning research. GSR is a variation of skin electrical resistance. It is determined by passing a weak current through the skin and measuring the change in current or by measuring the current generated by the body itself. GSR has been reported to be associated with emotion, attention and stress (Mohan et al., 2011). The relationship between GSR and attitudes, empathy and social interactions, especially with small groups, has been shown in Prokasy and Raskin (1973). In this study, using the latest achievements in modern medicine and physiology, through microcomputer interfaces and network communication technologies as well as virtual instrumentation, we will develop and design an Internet-based psychophysiological response testing and analysis system for creative learning, which will be used to record various physiological data and real-time performance of student participants during creative learning in real time. By analyzing these signals, this system can be used to investigate the relationship between different stress levels and performance on different creative learning tasks.

This paper focuses on the design of the system, its hardware and software and their respective implementations.

## The Design of the Hardware in the System

**FIGURE 1:**
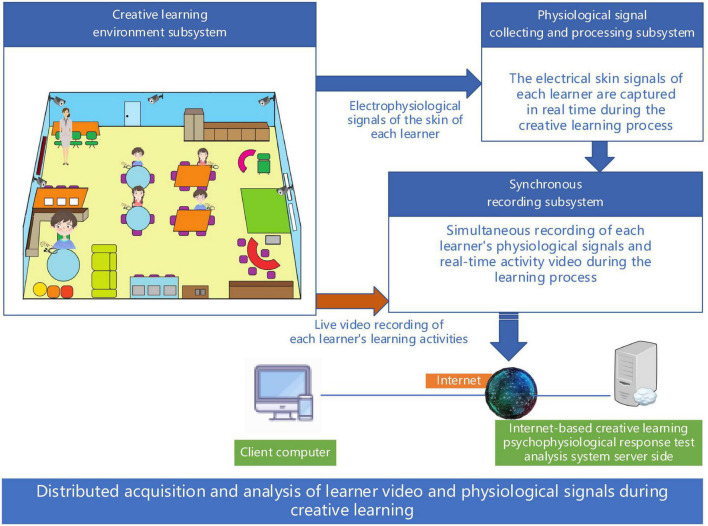
Components of the system.

The system mainly consists of three parts: creative learning environment subsystem, physiological signal acquisition and processing subsystem, and synchronous recording subsystem, which can be arranged according to the requirements of creative learning activities carried out and the conditions of the laboratory, as shown in Figure 1.

### Creative Learning Environment Subsystem

This subsystem is the main creative learning environment, including all kinds of hardware and software needed to carry out creative learning activities. The learning environment should support students whether they are working in small groups, pairs, or individually. High tables, low tables, round, and square tables—a variety is preferable. Anything hung in the classroom should be meaningful and support learning. Each space in the Creative Learning Environment sub-system should be carefully designed and organized to create a creative learning environment in which students can engage in creative learning and complete creative learning tasks.

**FIGURE 2:**
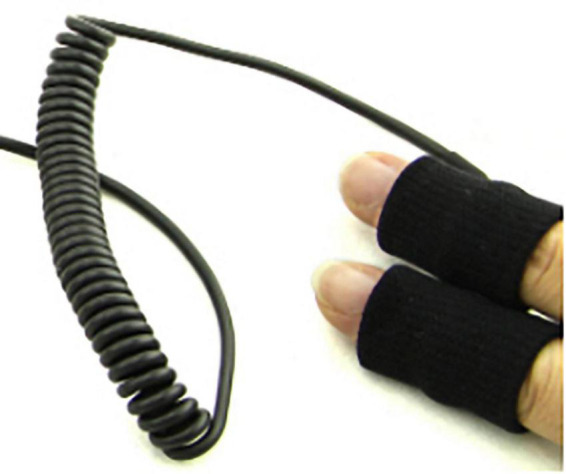
Galvanic skin response sensor worn on the learner’s hand.

Learners wear electrical skin sensors on their fingers (as shown in Figure 2), and as they complete creative learning tasks, their electrophysiological skin signals are transmitted to the physiological signal acquisition and processing subsystem. At the same time, a video recording of each learner’s creative learning process is synchronized by a camera and sent to the synchronized recording subsystem along with the learner’s skin electrophysiological signals.

### Physiological Signal Collecting and Processing Subsystem

**FIGURE 3:**
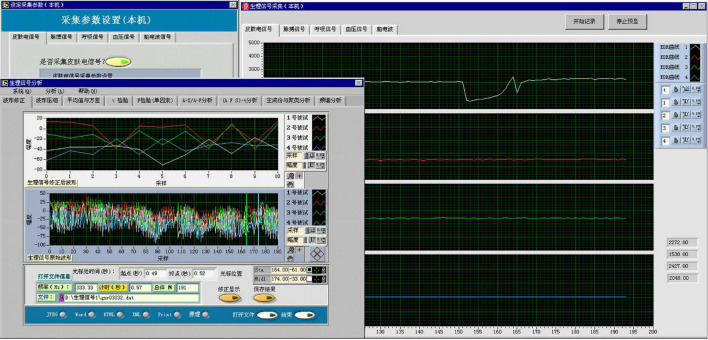
Physiological signal acquisition and processing program (software runtime interface).

The subsystem consists of software and hardware components. It is used to implement the analog-to-digital conversion of physiological signals, and to display, store, analyze and process these physiological signals. The software part is used for the collection, storage, analysis, and processing of the physiological signals (as shown in Figure 3). The hardware part of the subsystem consists of a collector, a multimedia computer and a printer. The UA301 data collector is used for the analog-to-digital conversion of physiological signals such as electrical skin responses. The multimedia computer installs software for collecting, analyzing and displaying physiological signals during the learner’s creative learning process. The printer is used to print the results of the analysis and processing.

### Synchronous Recording Subsystem

**FIGURE 4:**
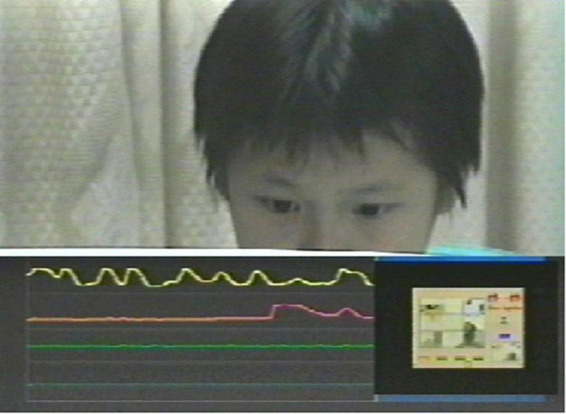
A simultaneous recording of a learner’s creative learning process, psychophysiological response signals, and facial expressions.

This subsystem mainly includes video monitors, video recorders, etc., which are used to record the creative learning process, various physiological signals of students and students’ expressions in real time and synchronously. Figure 4 shows a learner’s creative learning process, psychophysiological response signals, and facial expressions recorded by the synchronous recording subsystem during a creative learning session.

## The Software Design and Implementation in the System

The system is developed by means of virtual instrumentation technology, with LabVIEW2019 as developing tool on Windows (Ehsani, 2016; Shalgar and Bindu, 2022).

### Introduction to the Virtual Instrumentation and Its Developing Tools

Virtual instrumentation integrates computer hardware resources with instrumental hardware via software, resulting in the incorporation of the powerful computing and processing capabilities of the computer with the measuring and controlling capabilities of the instrumental hardware, thus greatly reducing the cost and volume of the instrumental hardware, and achieving data display, storage and analysis via software. Users can operate the computer with user-friendly graphic interfaces to collect, analyze, judge, display, and store the tested indexes. In the virtual instrumentation system, any user can easily alter, add or delete the functions and scopes of the system by modifying the software.

Among numerous virtual instruments developing software, LabVIEW by NI Company is a kind of graphic programing software designed for data collection, instrumental control, data processing, and data displaying. Compared with other software-developing environment, it possesses such obvious advantages as high developing efficiency, graphic programing, fast-generating models, modularized design, numerous graphic user interfaces, embedded database and toolbox, open structure, and good expandability.

### Functional Module Design of the Software

**FIGURE 5:**
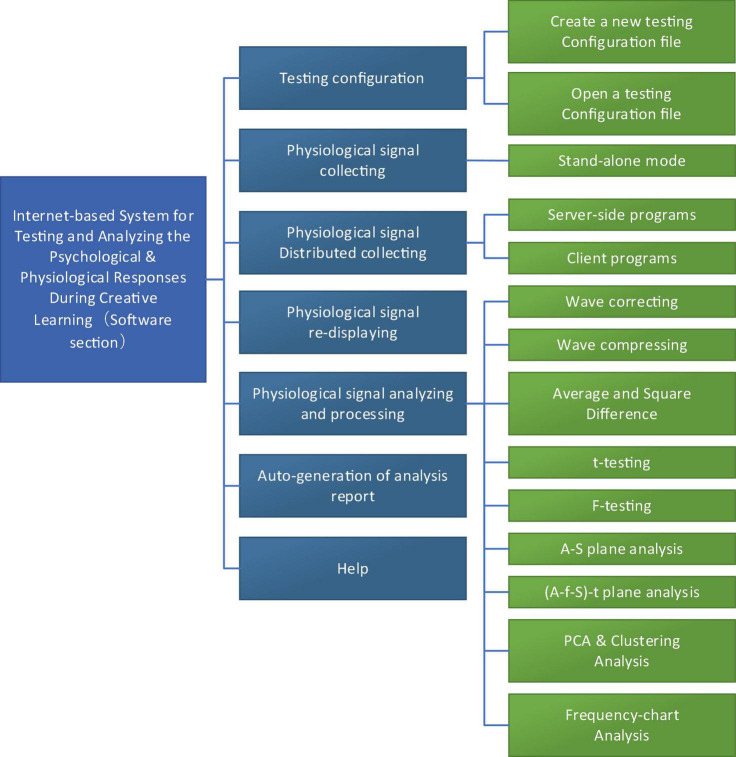
Functional modules of the software.

The system involves seven functional modules: testing configuration, physiological signal collecting, distributed physiological signal collecting, physiological signal redisplaying, physiological signal analyzing and processing, report generating and printing, help, as shown in Figure 5.

#### The Module for Testing Configuration

This module mainly achieves the testing configuration before collecting physiological signals, and generates the testing configuration report.

User can create a testing configuration file with the help of the guide and generate the file in Word format, including test number, test name, test purpose, parameters to collect, experimental object, path to store the physiological signal recording file, experimenter, testing date, and remarks.

User can collect data according to the default parameters, or collect data by opening existing configuration file.

#### The Module for Physiological Signal Collection

The module is used to real-time collect, synchronously display and real-time record physiological signals.

According to the settings in the testing configuration file, the physiological signals from the sensing system will go through the simulative-digital transformation with UA301 data collector. With the module, physiological signals can be collected, synchronously displayed, and stored in a hard disk if needed for further analysis.

#### The Module for Distributed Physiological Signal Collection

Adopting C/S mode, the module achieves the distributed remote collection of the Internet-based physiological signals with Data Socket protocol developed by NI Company.

Client end can define collecting parameters of the Server end (type of the physiological signals, collecting channel, sampling frequency and the amplifying times for the sampled signals), and the location to store the physiological signals of the Client end and the Server end, finally achieving the distributed remote collecting of the physiological signals via network.

#### The Module for Physiological Signal Re-displaying

The module is used to re-display the stored physiological signal recording files to analyze waves of physiological signals afterward, in combination with the synchronously recorded video contents. User can modify the displaying speed to fast re-display, slow re-display, or normally re-display (i.e., at the speed of signal collecting).

#### The Module for Analyzing Physiological Signals

The module mainly provides various means to analyze and process physiological signals, including wave correcting, wave compressing, average and square difference, *t*-testing, F-testing, A-S plane analysis, (A-f-S)-t plane analysis, Principal Component Analysis (PCA) and Clustering Analysis, Frequency-chart Analysis.

#### The Module for Generating and Printing Reports

The module is integrated with the above five modules to generate analysis reports in various formats such as Word or Excel and to store the analyzed results such as waves or tables in different formats or directly print the resulted various graphs, tables, and/or waves or to store each object on the screen in different graphic formats according to user’s requirements.

#### The Module for Help

The module is also integrated with the above modules, and required helps are available anytime.

### The Implementation of the Software

The following section will introduce the implementation process of the software by illustrating the server end of the module for distributed physiological signal collection.

The properly collection of physiological signals is the prerequisite to conduct the application study of physiological indexes and determines the subsequent analysis of these signals, therefore, the proper collection of physiological signals is the key to the system. The integration of virtual instruments with network technology to form the distributed networked the virtual instrument system is one direction of the virtual instrument system development. There are mainly two basic models to create Internet-based distributed remote virtual intelligent testing system: C/S model and B/S model. This system mainly adopts C/S model to achieve Internet-based distributed remote collecting and monitoring of physiological signals.

#### Description of the Function

The distributed physiological signal collecting and monitoring in the system is mainly for the remote computer to collect and monitor physiological signals at the server end via the network. The client end defines parameters for the server end to remotely collect physiological signals which can be stored in the server end or the local client end, or it simply monitors the waves of physiological signals being collected at the server end.

#### Flow Chart of the Program (Omitted)

*Implementation of the Programing*

The system adopts DataSocket of LabVIEW to achieve the Internet-based distributed remote collection of the physiological signals.

DataSocket (DS) provides a single interface which can be used by various programing languages and multiple types of data communication. There are two functions in LabVIEW to receive and distribute data: DataSocket Read and DataSocket Write. The program to distribute data automatically transforms user’s data into byte flow transferring on the network via DataSocket Write, while the data-receiving program reverts the byte flow into its original data type via DataSocket Read.

① The Program Design for the Server End

DS function adopts DSTP (DataSocket Transfer Protocol) developed by NI to communicate with DS server. In order to start the remote collection of physiological signals, it’s necessary to first determine the address of DS server, such as dstp://192.168.7.89/daqset, where daqset is data label used to determine the address of specific data item on DS server. In practical application, the address at the server end must be identical with the address where the Client needs to access so as to establish connection to achieve the remote collection and monitoring of physiological signals.

When the communication between the collecting program at the server end and DS server is established, the Client can begin to define the parameters for physiological signal collection at the server end. At this point the Client sends data to the Server via the function of DataSocket Write, while the server captures the data for parameter setting from the Client via the function of DataSocket Read.

② The Program Chart for the Server End

**FIGURE 6:**
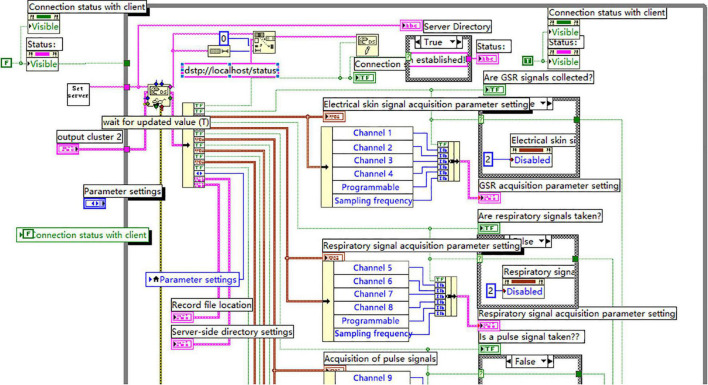
The chart program to real-time collect, display and store physiological signals at the server end (partially).

Figure 6 partly shows the program to real-time collect, display and store physiological signals. In the program, the secondary VI, i.e., SetServer, is designed to start DS server in order to establish the communication between the collection programs for the physiological signals at the server end with DS server.

## Conclusion

It has been shown that stress negatively affects creativity by reducing the demand for cognitive resources that are not available for creative thinking, and that people adopt simpler cognitive strategies that may undermine creativity (Byron et al., 2010). Some theories suggest that stress is positively related to creativity. Stress leads to creative thinking and motivates people to find solutions to problems (Nicol and Long, 1996; Anderson et al., 2004). If individuals are exposed to stress, they may use problem-solving strategies, which can increase creativity (Bunce and West, 1994). Stress can positively influence creativity by requiring creative solutions and providing cognitive stimulation and motivational stimulation for creative thinking (Deary, 1996; Anderson et al., 2004). Some researchers have proposed an alternative scenario for the relationship between stress and creativity (Avey et al., 2012), in which stress is curvilinearly related to creativity (Yerkes and Dodson, 1908). Activation theory (Gardner, 1986) assumes that stress can increase performance up to a certain threshold, but too much activation can limit performance, especially for complex tasks, such as creative tasks. Individuals are likely to be most creative at moderate levels of activation. Moderate levels of activation increase task engagement and optimize the use of cognitive resources by reducing negative effects (Gardner, 1986; Baer and Oldham, 2006). Conversely, too little or too much activation may lead to lack of engagement and cognitive interference that may interfere with performance on cognitively demanding tasks. Given that cognitive, emotional, and behavioral engagement are important processes for creativity (Drazin et al., 1999), moderate activation leads to maximum creativity (Byron et al., 2010). The system developed in this study is capable of dynamically acquiring and analyzing psychophysiological data of students during creative learning, while measuring cognitive, behavioral, psychological, and physiological indicators of learners during the creative learning process. Extending the study of creative learning processes and performance to the physiological level and providing advanced support environments, measurement tools, and analysis methods, it breaks through the limitations of creative learning research in collecting information on learners’ cognitive, psychological, and emotional responses only through questionnaires and self-assessments. Using this system, the impact of learners’ psychophysiological responses to creative learning tasks during creative learning can be studied, for example it is used to study the changes in students’ psychophysiological states during the process of solving real-life inferior problems, and to provide teachers with assistance in designing interventions that can effectively enhance students’ real-life problem-solving and creative skills.

## Data Availability Statement

The original contributions presented in the study are included in the article/supplementary material, further inquiries can be directed to the corresponding author.

## Ethics Statement

The individual(s) provided their written informed consent for the publication of any identifiable images or data presented in this article.

## Author Contributions

The author confirms being the sole contributor of this work and has approved it for publication.

## Conflict of Interest

The author declares that the research was conducted in the absence of any commercial or financial relationships that could be construed as a potential conflict of interest.

## Publisher’s Note

All claims expressed in this article are solely those of the authors and do not necessarily represent those of their affiliated organizations, or those of the publisher, the editors and the reviewers. Any product that may be evaluated in this article, or claim that may be made by its manufacturer, is not guaranteed or endorsed by the publisher.
